# Teen-dating violence: conception of adolescents in a Brazilian metropolis

**DOI:** 10.5249/jivr.vo112i2.1528

**Published:** 2020-07

**Authors:** Stella R. Taquette, Claudia Leite Moraes, Luciana Borges, Simoni Furtado

**Affiliations:** ^*a*^ Medical Sciences College, State University of Rio de Janeiro (UERJ), Rio de Janeiro, Brazil.; ^*b*^ Institute of Social Medicine, State University of Rio de Janeiro (UERJ), Rio de Janeiro, Brazil.; ^*c*^ Nursing School, State University of Rio de Janeiro (UERJ), Rio de Janeiro, Brazil.

**Keywords:** Teen dating violence, Gender, Exposure to violence, Focus group, Qualitative method

## Abstract

**Background:**

Teen-dating violence (TDV) can lead to health problems for those involved, especially females, including homicides and is predictive of intimate partner violence in adulthood.

**Methods:**

To analyze the adolescents' perception of definitions, justifications and consequences of TDV, a qualitative study through 13 focus groups with 132 high school students from public and private schools of Rio de Janeiro city, including 70 girls and 62 boys was conducted. We followed a guide with questions about interpersonal violence and participants completed a self-administered questionnaire on socio-demographic data. The data analysis included reading and comprehension of the textual data; coding of the reports according to the emerged categories; identification of the meanings attributed by the subjects to the questions raised; comparative dialogue with literature; and elaboration of interpretative synthesis.

**Results:**

Most adolescents affirmed that TDV is not justifiable. However, in practice, they found it acceptable in certain situations. The narratives of the boys were based on the role of the perpetrator and the girls on that of the victim. Violence occurs when the man feels his power challenged and is influenced by situations of violence experienced in his own family as victims or witnesses. The TDV consequences are for the women and include, in addition to physical and psychological damage, relational problems in other spheres such as family, friends and school. The experience of violence was more common among public school students.

**Conclusions:**

The adolescents’ narratives reflect the gender patterns of society in which violence results from inequality of power. The data of this study offer subsidies to policies on TDV prevention and its consequences. They can contribute to training primary care professionals to identify on clinical consultations signs and symptoms of violence and to develop interventions to reduce the health problems of victims.

## Introduction

Teen dating violence is that which occurs between partners involved in a romantic relationship.^1^ It is an event of significant prevalence in several parts of the world.^2-7^ It can have immediate and late health consequences, with women being the most frequent victims of serious injuries.^8-10^ TDV is often not perceived by those involved or valued by society, and is a predictor of violence between intimate partners in adulthood. ^11,12^ Several population-based studies indicate the correlation between TDV and mental health problems, including depression, anxiety, and alcohol/drug abuse. ^13-18^ Sexual behaviors that increase the risk of sexual transmitted infections (STI) and AIDS and low academic performance are also pointed out as negative consequences of TDV.^19-21^ In addition, there is evidence that violence in other settings, such as family and neighborhood, may be related to TDV, ^22,17^ corroborating the idea brought by some authors that violence occurs in a circular way and in the different contexts of adolescent socialization.^7,23-26^

In different parts of the world TDV has been treated as an important public health problem and one of the main issues to be faced regarding adolescent health. ^27-30^ As a consequence, several policies and programs of action have been implemented, especially in schools.^31-34^ However, the issue is still little discussed in developing countries.^35-37^

This study hopes to add knowledge of TDV in the context of a large urban center in a developing country where the frequency of TDV is high. The severity of health damage it causes and its association with intimate partner violence in adulthood is also high. The authors have carried out extensive literature review in the last 5 years on the subject and found few studies of qualitative nature.^26^ This study intends to fill this gap. It intends to apprehend, from the adolescents’ perspective, what do they consider dating violence, why does it occur and what are its consequences. Knowing the vision of the individuals involved is fundamental for the elaboration effective actions for confrontation.

## Methods

In this field of research, most of the produced knowledge is of a quantitative nature and corresponds to prevalence of TDV estimated and associated factors. Therefore, we opted for the qualitative method with focus group discussion, that is a valuable resource for understanding the process of building perceptions, attitudes and social representations of human groups. 

The research was conducted with second year high school students, of both sexes, in public and private schools of Rio de Janeiro city, Brazil. The election of this target group was due the greater probability that they have dating experience and still are in the teenage group. 

**Fieldwork**

The data were collected during 2016 school year with students, parents, and schools' authorities authorization. The focus group followed a script that included the following issues: what they consider dating violence, justification for its occurrence and its consequences. At the end of meeting, each participant filled a small questionnaire with their socio-demographic data. The groups were recorded in audio and later transcribed. The sample criterion was the saturation of contents with the guarantee of a balance between the number of participants from public and private schools and of both sexes. 

**Data analysis**

We performed data analysis through the following steps:^38^ reading and rereading the textual data to get a sense of the whole, of the convergences and divergences of thoughts in order to identify the central ideas; classification of the central ideas in categories and cutting and pasting of the text according to them; identification of the meanings attributed by the subjects to the questions raised; comparative dialogue with scientific data from other studies; and elaboration of interpretative synthesis.

**Ethical aspects**

The Research Ethics Committee of the State University of Rio de Janeiro on 09/18/2015 approved the research and all participants and their legal guardians signed an informed consent form. 

## Results

We conducted 13 focus groups in six schools, three privates and three publics, chosen by raffle, totaling to 132 students, 50% of each segment. The female students represented a discrete majority, 55% of total. Five groups were composed only of male members, five females and three mixed with the intention of increasing the variability of the study. Socio-demographic and family profile, according to participant’s sex and school-administrative characteristics, are presented in Table 1.

**Table 1:** Study sample profile according to sociodemographic and family aspects, stratified by sex of the participants and school-administrative characteristics.

Variables		Sex				School-administrative characteristics			
		Girls		Boys		Public		Private	
		n	%	n	%	n	%	n	%
**Age Group**	15 to 17 years old	53	73	41	68	36	54	58	88
	18 to 21 years old	17	23	19	32	29	44	7	10
	> 21 years old	1	2	0	0	1	2	0	0
	Uninformed	1	2	0	0	0	0	1	2
	**Total **	**72**	**100**	**60**	**100**	**66**	**100**	**66**	**100**
**Ethnicity**	White	37	52	24	40	15	23	46	70
	Non-White	35	48	35	58	51	77	19	28
	Uninformed	0	0	1	2	0	0	1	2
	**Total **	**72**	**100**	**60**	**100**	**66**	**100**	**66**	**100**
**Family Income**	≤ 3 minimum wage	29	40	28	46	42	63	15	23
	> 3 minimum wage	36	50	24	40	18	27	42	64
	Uninformed	7	10	8	14	6	10	9	13
	**Total **	**72**	**100**	**60**	**100**	**66**	**100**	**66**	**100**
**Family Organization**	Single parent	30	42	15	25	25	38	20	30
	Two parents	35	49	42	70	33	50	44	67
	Other	7	9	3	5	8	12	2	3
	**Total **	**72**	**100**	**60**	**100**	**66**	**100**	**66**	**100**
**Higher Parental Schooling**	Incomplete Elementary School	8	11	3	5	11	17	0	0
	Complete Elementary School to Incomplete High School	3	4	5	8	6	9	2	3
	Complete High School to Incomplete College Education	22	31	23	39	35	53	10	15
	Complete College Education or	37	51	26	43	11	17	52	79
	Uninformed	2	3	3	5	3	4	2	3
	**Total**	**72**	**100**	**60**	**100**	**66**	**100**	**66**	**100**

In the analysis of the textual data, we classified them into two general categories: concept and justification of violence and their consequences in adolescents’ lives. We observed differences between the narratives of girls and boys, as well as between students of public and private schools as we can see below in the results.

**Concept and justification of violence**

The conceptions of violence, in theory, were coincidental in both sexes and types of school. Everyone maintained that any type of aggression, verbal, physical or sexual is violence. Most students reported that the relationship must end when violence occurs. 

However, in the course of the debate, this conception was detailed, qualified and sometimes contradicted. "Nothing justifies violence on a relationship": this was the predominant idea in the groups. However, students' narratives show that sometimes the intimacy of a relationship allows some aggressive attitudes that become "acceptable." They recognize that they can "lose their mind" in certain situations, as in cases of betrayal. According to their reports, the most common type of TDV is verbal/psychological, which manifests itself in the form of humiliations, name-calling and attacks on self-esteem. Another reason often suggested for the practice of TDV was the experience of violence in the family as direct victims or witness violence in the family.

**The girl’s narratives**

We observed that girls placed themselves in the victim’s position, unlike the boys who spoke as aggressors. For the private schools’ female adolescents, dating violence was the way for boys to maintain control in a relationship, because they had the need to impose themselves and to demonstrate superiority. It happens because men feel like owners of their partners. They determine what their partners can or cannot do or whom to be friends with, what clothes to wear, and which places to go to. They report that boys who reaffirm traditional gender roles engage more in acts of violence.

-“I think that he can feel threatened, he may feel that his masculinity is at stake. So, he begins to grow in a physical way, an aggressive type.” (PUBLIC SCHOOL GIRL)

On the other hand, some female adolescents do not see as violence that kind of control, on the contrary, they even feel loved when their partner prevents or prohibits them from doing something or wearing certain clothes. They place themselves in a submissive role and feel guilty and/or deserving when they are victims of aggression:

“I have nothing to complain about him preventing me from doing things, complaining about clothing. And because he was like that, I liked him very much.” (PRIVATE SCHOOL GIRL) 

In the public-school groups, the female adolescents spoke more intensely about other forms of violence than domination by their boyfriends. The reports of physical and sexual aggression were more frequent than in private schools, leaving the impression that these types of violence are more experienced and/or more explicit by this social stratum. Some do not realize that they are suffering TDV and others, although find wrong any type of aggression, in a context of partner infidelity, they are capable of attacking both the boyfriend and the third person. It has been pointed out by some female students that certain teen girls experience dating violence because they do not assess who they are relating with. They criticized the female adolescents who in the first date have sexual intercourse and later end up being victims of violence, inferring a causal relation between the two events. These teenagers who "give in" soon do so without knowing the partner well and therefore might be victims of violence.

**The boys’ narratives**

The boy’s justifications are similar to those described by the girls; however, they reported spontaneously in the perpetrator’s role. They demonstrated that it was unbearable for a man to have no authority in the relationship. Sometimes, the man hits to show that he is not a coward and that he is the legitimate holder of power. 

Their view is that they are stronger and their emotions as uncontrollable. Therefore, faced with a situation in which they were contradicted, it is natural to react violently because they have feelings they cannot control. 

-“It’s complicated because men’s emotions come much stronger than women’s in those situations.” (PUBLIC SCHOOL BOY)

For several boys in the public school, it is normal to hit lightly the woman from time to time, and this is not violence. In their view, the girls have more arguments in a disagreement and by not being able to “win” in a discussion, they appeal to the aggression to assert their will.

In case of betrayal, the reaction demonstrated by some was violent. The loss of confidence gives the boy right to assault his girlfriend. The same reaction can occur when they consider that the partner did something wrong. They are the ones who have power to determine what is or not acceptable. 

- “I think it's normal, because if a woman acts badly sometimes, she deserves to be beaten. It’s not beat up, it’s slap a little bit. She’s wrong. She knows she has to be spanked.” (PUBLIC SCHOOL BOY)

In private schools, although the boys recognize that TDV happens because the man wants to maintain control in the relationship, they do not think that it is correct. They showed fewer tendencies to explain violent reactions to a betrayal than those of the public schools. When asked how they would react to a situation of explicit betrayal, they would end the relationship immediately, without engaging any violence to the partner. According to them, it is preferable to step away from the person than to attack her. Aggression is regarded as “primitive” behavior. 

On the other hand, public school students spoke more often about situations of violence experienced in the family and in the neighborhood. Violence in the family was also reported by them as a role model. According to reports, seeing a mother being beaten up by her partner makes children and adolescents learn that this is man’s natural behavior. However, there were those who disagreed and argued the opposite, that this situation would make them learn not to be violent with their partners. 

**Consequences of TDV in adolescents’ lives**

The students’ narratives highlighted the consequences of violence for women and very little for men. The man is never a victim of violence, because he is physically stronger. But for women, the damages range from social isolation to death, passing through the possibility of becoming a lesbian due to the aggressions suffered. Disillusionment, grief, and lack of affection experienced in a violent relationship can lead girls to seek same-sex relationships. Both boys and girls suggested this understanding. In their view, same-sex relationships are more egalitarian. Therefore, there chance of disagreements and violence is smaller. 

**The girls’ narratives**

The theme was very feelings mobilizing in the female groups. Almost all the meetings culminated in dramatic testimonies of some girls who experienced TDV, with serious consequences in their lives. They also mentioned that almost all of them have had abusive relationships. There were students who were moved and wept during the meeting.

Women who are in abusive relationships become isolated, lose their freedom, and alter behavior to adapt to their boyfriends’ wants. They have their self-esteem reduced, feel guilty for the aggression they suffer and therefore self-flagellate frequently. The teenager accepts any imposition from her boyfriends. By the partner’s action, they change the way of being, dressing, and interaction with other people. 

“It changes even you. I put on makeup every day to come to school. And last year I didn’t do that. I didn’t do anything. I looked like an animal.” (PRIVATE SCHOOL GIRL)

The consequences extend to school life and to physical and mental health. Dating violence causes a separation from family and friends and various psychological disorders. The most serious consequence pointed out by the students was feminicide. This possibility was raised in all groups in cases where the woman wanted to end the relationship, and the partner did not accept.

**The boys’ narratives**

The main characteristic perceived was that they do not see themselves as dating violence victims. They think girls are physically more fragile and are not able to hurt them. However, they pointed out that when they happen to be victims of aggressions that they consider relevant, they have no one to turn to, because no one recognizes or values their complaints. 

According to our interlocutors, when a man is violent with his partner and she does not react or only cries, it means that he will easily assault her again. On the other hand, when she reacts, the chance of a new aggression will be smaller. Therefore, it seems to be the woman’s responsibility to stipulate this limit in the relationship. 

-“From the first slap that the man gives the woman and she doesn’t like it and talks seriously, the man will think twice. Now, if she doesn’t say anything, just cry, then after 10 minutes passed, they talk again as a couple, then it happens all over again.” (PUBLIC SCHOOL BOY)

## Discussion

In the first category, like Reidy et al ^39^ in a survey with 589 male adolescents, the reports of our participants make it clear that dating violence occurred as a way of reasserting male dominance in cases where boys have their power threatened. Violent action is one way for them and to confirm their masculinity. The feeling of possession and power of a man over woman is based on the patriarchal culture and has influenced society up to present day. ^40^

This relation of domination that justified violence in some situations was often considered normal in dating and even exchange of slaps and curses are considered as jokes.^41^ This dynamic has also been ascertained in relationships of adolescents from other developing countries, such as Egypt, where early marriage of girls is little recognized by them as gender violence. ^42^ On the other hand, there is evidence that adolescents who perceive violence in a relationship engage less in these violences and have a more conservative attitude toward sexual activity.^43,44^

The reports of adolescents about the devaluation of women who initiate intimate relationships early reflect the hegemonic patterns of gender. In this model, it is up to women to be submissive and sexually modest to characterize their decency and thus not be violated. On the other hand, disagreeing with what the girls described, a study conducted in Brazil with female adolescents younger than 15 years found that being a victim of violence contributes to the early and unprotected onset of sexual activity.^45^ That is, suffering violence is what can lead to the early onset of sexual activity and not the opposite.

The boys demonstrated in their narratives that they rigidly follow the hegemonic gender roles in society. These data corroborate Cecchetto et al^46^ research developed with male students from ten Brazilian capitals. The meanings attributed to the phenomenon of dating violence correspond to the expectations of its performance in affective-sexual relations. Within this same logic, violence is justified in cases of jealousy and betrayal as corroborated by other studies.^47,48^

Family influence on adolescent behavior is common finding for several studies.^49,50^ The child who grows up in an environment in which parents attack each other, learns to do so. They reproduce the model of behavior experienced at home.^49^ Alleyen-Green et al ^51^ identified associations between TDV, risky sexual behavior, and poor involvement of biological parents in their children's lives. On the other hand, it seems that adolescents with a good family relationship engage less in dating violence.^52^ In Sweden, a student survey revealed a high frequency of TDV associated with the absence of both parents in the family.^2^ Cascardi^53^ found a similar result, evidencing that TDV is related to childhood mistreatment. These factors, coupled with substance abuse and violence in the social environment of the neighborhood, were evidenced by Reyes et al ^25^ as associated with TDV. The association between lack of school support, intrafamily violence and TDV was also suggested by Earnest et al.^54^ Diaz-Aguado^55^ study of 4147 boys demonstrated that for them aggression is a means of resolving conflicts as witnessed among adults in their families.

In the second category, the reports of the students show that consequences of dating violence are more harmful for women. Studies highlight the greater frequency of damage to them.^3,39^ Men who are stronger do not feel affected by their partners’ aggressions and are more likely to laugh at physical violence suffered. ^10,50,56^

According to our interlocutors, one of the consequences for women victims of violence is to have relationships only to women. These notes oppose studies that indicate that violence among sexual minorities is greater. Luo et al^57^ in a youth risk behavior survey found that sexual minorities have significantly increased rates of intimate partner physical violence compared to heterosexuals. Martin-Storey,^58^ in a survey with a sample of 12,984 adolescents, found a higher prevalence of TDV among the young sexual minority.

The serious consequences of TDV pointed out by our interlocutors, including social isolation, school difficulties, depressive symptoms with suicidal feelings, alcohol and drug abuse, risky sexual behavior, and the possibility of death are corroborated by several studies.^10,13,59^

In summary, we can emphasize in the narratives of the adolescents of our study that TDV is a result of the gender patterns of the society in which violence results from inequality of power. It has a circular character, that is, it is experienced in other contexts. They perceive more the consequences for the women that include, in addition to physical and psychological damage, relational problems in other spheres such as family, friends and school. The data of this study offer subsidies to policies on TDV prevention and its consequences as well as in promotion of adolescence health in order to reduce the problems of victims.

Another relevant place for preventive action is the school, which is the scenario of more intense and prolonged coexistence of the adolescent population, propitious to actions to prevent adult aggravations and of health promotion, where anti-violence pedagogical proposals based on gender equality, respect to diversity and human rights can and should be developed. 

We emphasize as a limitation of the study that qualitative data are not generalizable and should be contextualized in the social and political space where they were collected. However, they are relevant to the understanding of similar contexts. 
